# How Can We Support the Use of Systematic Reviews in Policymaking?

**DOI:** 10.1371/journal.pmed.1000141

**Published:** 2009-11-17

**Authors:** John N. Lavis

**Affiliations:** 1 McMaster Health Forum, McMaster University, Hamilton, Ontario, Canada; 2 Centre for Health Economics and Policy Analysis, McMaster University, Hamilton, Ontario, Canada; 3 Department of Clinical Epidemiology and Biostatistics, McMaster University, Hamilton, Ontario, Canada; 4 Department of Political Science, McMaster University, Hamilton, Ontario, Canada

## Abstract

John Lavis discusses how health policymakers and their stakeholders need research evidence, and the best ways evidence can be synthesized and packaged to optimize its use.

**Summary Points**

- Policymakers need many types of research evidence—synthesized and packaged for them—and the use of this evidence supported in multiple complementary ways. Stakeholders who seek to influence the policymaking process have the same requirements.
- Policymakers and stakeholders need many types of systematic reviews. For example, reviews of qualitative studies can help to identify alternative framings of the problem, to understand how or why a policy or program option works, and to appreciate stakeholders' perspectives on particular options.
- Policymakers and stakeholders now have access to many review-derived products: (1) summaries of systematic reviews highlighting decision-relevant information; (2) overviews of systematic reviews providing a “map” of the policy questions addressed by systematic reviews and the insights derived from them; and (3) policy briefs drawing on many systematic reviews to characterize a problem, policy or program options to address the problem, and implementation strategies.
- A range of activities are being undertaken to support the use of reviews and review-derived products in policymaking, all of which warrant rigorous evaluation.
- Future challenges include: (1) examining whether and when any apparent duplication of efforts occurs in the production of review-derived products at the international level; and (2) scaling up activities that are found to be effective in supporting the use of reviews and review-derived products in policymaking.

## Introduction

In the last few years the landscape has changed dramatically for policymakers seeking to use research evidence in the policymaking process. The landscape has also changed for the many stakeholders seeking to use research evidence to influence the policymaking process. The task once seemed overwhelming given the dearth of synthesized research evidence on the “big”, typically multifaceted, questions that matter to policymakers and stakeholders [1],[2]. Now it isn't uncommon for these groups to find dozens of systematic reviews that address the governance, financial, and delivery arrangements within health systems that can determine whether a cost-effective program, service, or drug reaches those who need it. For example, teams of African policymakers, stakeholders, and researchers drew on 30 reviews for what at first glance seems a straightforward question: how to support the widespread use of artemisinin-based combination therapy (ACT) to treat uncomplicated falciparum malaria. The review of qualitative studies of people's views about and experiences with medicine sellers provided insights that were as central to the process as reviews of the effectiveness of a particular ACT formulation or the home-based management of malaria [3]–[5].

For policymakers and stakeholders the challenge in using research evidence has shifted from making the best possible use of local studies to: (1) finding systematic reviews that address their many questions related to the policy issue at hand; (2) deriving insights from the reviews for a particular context (which may differ from where the studies included in the review were conducted); and (3) combining these insights with the insights from local data and studies and from local tacit (“how to”) knowledge and other forms of knowledge [1],[6]–[8]. Policymakers and stakeholders then need many types of systematic reviews, for these reviews to be packaged in different ways in order to facilitate their use in policymaking, and more generally for their use of the reviews to be supported in multiple complementary ways. The same holds true for health system managers, including those working in hospitals, nongovernmental organizations, and many others settings. In some countries these managers are counted as policymakers, and in others they are counted as stakeholders. In all countries they are decision makers in their own right. Much of what follows applies to health system managers as well.

## Need for Many Types of Reviews

Policymakers and stakeholders need many types of reviews to inform any given policymaking process (Table 1). For example, reviews of observational studies can help to establish the magnitude of the problem (or the factors that contribute to it), characterize the harms and key elements of policy and program options for addressing the problem, and identify potential barriers to implementing a preferred option [9],[10]. Reviews of qualitative studies can help to identify alternative framings of the problem, understand how or why a policy or program option works (particularly if local adaptation is being considered), appreciate stakeholder's views about and experiences with particular options, and identify potential barriers to implementing a preferred option [11]–[13]. Reviews of effectiveness studies can help to characterize the benefits and sometimes the harms of each option being considered [14]. And finally, reviews of economic evaluations can help to characterize the cost-effectiveness of options [15]. Policymakers and stakeholders can find increasing numbers of all of these types of reviews.

**Table 1:** Examples of the types of systematic reviews needed in different steps in the policymaking process.

Steps in a Policymaking Process	Sub-Steps that Involve Acquiring Data and/or Research Evidence	Examples of the Types of Systematic Reviews That Can Be Acquired
**Defining the problem**	Identifying indicators to establish the magnitude of the problem (or the factors that contribute to it)	Reviews of observational studies (e.g., administrative database studies, community surveys)
	Making comparisons (over time, across settings or against plans) to establish the magnitude of the problem (or the factors that contribute to it)	Reviews of observational studies (e.g., administrative database studies, community surveys)
	Highlighting alternative framings of the problem to assist with mobilizing support among different groups to address the problem	Reviews of qualitative studies that examine stakeholders' views about and experiences with the problem (e.g., studies in which narrative data are collected from individual or groups of “informants” through interviews, focus groups, participant observation, or from documents)
**Assessing potential policy and program options**	Identifying policy and program options that could affect the problem (or the factors that contribute to it)	(Frameworks embedded in) Reviews or overviews of systematic reviews of any type if frameworks were used to organise the search for, and presentation of, research evidence (as well as theories and frameworks that are the focus of articles/reports in their own right)
	Characterizing the positive effects (benefits) of each policy option	Reviews of effectiveness studies (e.g., randomized controlled trials, interrupted time series)
	Characterizing the negative effects (harms) of each policy option	Reviews of effectiveness and/or observational studies
	Characterizing the cost-effectiveness of policy options	Reviews of economic evaluations
	Identifying the key elements of complex policy options (to facilitate local adaptation if necessary)	Reviews of qualitative studies that examine how or why interventions work and/or reviews of observational studies
	Characterizing stakeholders' views about and experiences with the policy options	Reviews of qualitative studies that examine stakeholders' views about and experiences with particular options
**Identifying implementation considerations**	Identifying potential barriers to implementation at the level of patients/consumers, health workers, organizations, and systems	Reviews of observational studies and/or reviews of qualitative studies
	Characterizing the effects of appropriately targeted implementation strategies	Reviews of effectiveness studies

Of course the insights derived from these reviews must compete with many other factors in the policymaking process, such as institutional constraints, interest group pressure, citizens' values, and other types of information like policymakers' past experiences [16]. But some of these types of reviews can provide helpful ammunition in a fight over problem definition or helpful background for a discussion with stakeholders about a problem or about possible policy and program options to address it. Moreover, the strategic use of reviews during “windows of opportunity” created by political events, such as the election of a new government or an interest group pressure campaign, can help to push some problems or options higher or lower on the agenda [17]. Systematic reviews can also be drawn upon to develop a monitoring and evaluation plan when there is substantial uncertainty about what can be expected from a policy or program.

## Growing Availability of Review-Derived Products

Policymakers and stakeholders now have access to at least three types of review-derived products: (1) summaries of systematic reviews that highlight decision-relevant information; (2) overviews of systematic reviews that provide a “map” of what policy questions have been addressed by systematic reviews and where additional reviews are needed and that derive insights from these reviews; and (3) policy briefs that draw on many systematic reviews to better understand a problem, policy or program options to address the problem, and possible implementation strategies for these options (Table 2). Some summaries add significant value to a review by highlighting key findings, evaluating the review's quality [18],[19], grading the strength of evidence contained in it [20], identifying local applicability and equity considerations [21]–[23], and/or providing commentaries by select users of the reviews. Identifying local applicability considerations is particularly important for reviews that address governance, financial, and delivery arrangements in health systems because the lessons learned from these reviews are likely to be context-sensitive [1],[24]. One key challenge lying ahead will be to ensure that summary-production processes keep up with the review-production/updating processes, which should include working through whether and when an apparent duplication of effort in the production of these summaries at the international level is problematic, and not simply the result of experimentation or appropriate targeting to particular audiences and contexts.

**Table 2:** Examples of review-derived products targeted at policymakers and stakeholders.

Type	Goal	Examples	
		Sponsor/Initiative	Systematic Reviews Included
**Summaries of systematic reviews**	**Summarize systematic reviews in order to: (1) allow policymakers to identify the take-home messages from systematic reviews that address their policy question (or elements of their policy question); and (2) (occasionally) add value to a review by evaluating its quality, grading the strength of evidence contained in it, identifying local applicability and equity considerations, and/or providing commentaries by select users of the reviews**	ACC Policy Liaison Initiative	Reviews of policy-relevant reviews, which are typically health system interventions, provider behaviour-change interventions, and consumer-targeted reviews [29]
		DARE	Reviews of the effects of health or health-system interventions
		Effective Health Care Research Programme Consortium	Same as ACC but with a particular focus on LMICs
		Evidence Aid	Same as DARE but with a particular focus on natural disasters and other health care emergencies (originally done in response to the 2004 tsunami and now updated in response to the H1N1 pandemic) [30]
		Health Knowledge Network of the CC&CRG Evidence Bulletins	Reviews of the effects of consumer-targeted reviews
		Health-evidence.ca	Reviews of the effects of public-health interventions
		Reproductive Health Library	Reviews of the effects of reproductive-health interventions, with a particular focus on LMICs
		Rx for Change	Reviews of the effects of prescribing-related interventions (and provider behaviour-change interventions more generally)
		SUPPORT	Same as ACC but with a particular focus on LMICs
**Overviews of systematic reviews**	**Systematically and transparently identify, select, appraise, and synthesize systematic reviews that address the broad array of research questions in a given domain in order to: (1) allow policymakers to identify systematic reviews that address their policy question (or elements of their policy question) and the take-home messages from these reviews; (2) allow policymakers to identify gaps in coverage by existing systematic reviews that will need to be filled through policymakers' own efforts to review research studies or through systematic reviews that they commission**	IDEAHealth	Reviews of the effects of health system financing mechanisms [31], human resource interventions, [32],[33], and interventions to reduce maternal and child mortality [34],[35], with a particular focus on LMICs
		SUPPORT	Reviews of the effects of interventions to support the delivery of cost-effective interventions in health systems and in maternal and child health, with a particular focus on LMICs [36]
		Cochrane Collaboration's EPOC review group	Reviews of the effects of provider behaviour-change interventions [37]
		Cochrane Collaboration's CC&CRG review group	Protocol for a review of the effects of consumer-targeted interventions [38]
**Policy briefs**	**Systematically and transparently identify, select, appraise, and synthesize systematic reviews, research studies, and context-specific data in order to address all elements of a policy question in order to: (1) allow policymakers to define the underlying problem, characterize policy and program options to address the problem, and identify implementation considerations; (2) allow policymakers to identify gaps in coverage by existing systematic reviews, studies, and context-specific data that will need to be filled**	Health Evidence Network/European Observatory on Health Systems and Policies	Reviews that inform problem definition, policy option characterization, and policy implementation-strategy characterization at the regional level, with a particular focus on the European Region
		EVIPNet	Reviews that inform problem definition, policy option characterization, and policy implementation-strategy characterization at the country level, with a particular focus on countries in Africa, Asia, and the Americas with formally established evidence-to-policy partnerships (EVIPNet)

ACC, Australasian Cochrane Centre; CC&CRG, Cochrane Consumers and Communication; DARE, Database of Abstracts of Reviews of Effects; EPOC, Effective Practice and Organization of Care; EVIPNet, Evidence-Informed Policy Networks; IDEAHealth, International Dialogue on Evidence-Informed Action to Achieve Health Goals in Developing Countries; LMICs, low- and middle-income countries; SUPPORT, Supporting Policy Relevant Reviews and Trials.

Overviews of systematic reviews can add value in some of the same ways as summaries of systematic reviews, albeit with a greater emphasis on breadth of coverage (e.g., the range of policy and program options examined) than depth of coverage (e.g., detail about what is known about any one option). Policy briefs, on the other hand, start with a policy issue, not with the reviews that researchers happen to have produced. The few current producers of policy briefs (as we have defined them in Table 2) have differed in their jurisdictional focus (e.g., whether the effort to contextualize the research evidence focuses on a country or a whole region) and in whether the briefs are seen as an end in themselves or as an input to one or more “deliberative dialogues.” Such dialogues typically involve convening one to two dozen policymakers and stakeholders to work through a policy issue, drawing on both the policy brief and their own and others' tacit knowledge about the issue.

## Activities That Could Support the Use of Systematic Reviews

A range of activities are being piloted to support the use of reviews and review-derived products in policymaking (Table 3) [25],[26]. Few evaluations of the effectiveness of these activities have been undertaken; however, a group led by researchers at the Melbourne School of Population Health is registering a title for a systematic review on this topic with the Cochrane Collaboration. All that is available to inform decisions about the relative emphasis to give to these options are systematic reviews of the factors that influence the use of research evidence in policymaking [2],[27],[28]. A small number of factors emerged in these reviews with some frequency:

- Interactions between researchers and policymakers increased (and a lack of interactions decreased) the prospects for using research evidence, particularly when the interactions were based on informal relationships;
- Timeliness increased (and a lack of timeliness decreased) the prospects for research use; and
- Accordance between research evidence and the beliefs, values, interests or political goals, and strategies of policymakers and stakeholders (or when political positions had not yet been taken) increased (and discordance decreased) the prospects for using research evidence.

**Table 3:** Examples of activities to support the use of systematic reviews and review-derived products by policymakers and stakeholders.

Approach	Examples of Activities
Promoting a climate that supports the use of reviews and review-derived products in policymaking processes	Citing examples from the past or from other jurisdictions where the use of reviews made the difference between policy success and policy failure
	Conducting an audit of policy documents to identify whether and how existing systematic reviews were cited
	Modifying policymaking processes to give an explicit role for systematic reviews, however, this is likely possible only for “routine” decisions like coverage decisions about prescription drugs and other “technologies” [39],[40]
Producing reviews and review-derived products that address high-priority policy issues	Undertaking priority-setting processes that identify short term (1–6 mo) requirements for review-derived products, medium-term (6–18 mo) requirements for systematic reviews, and long-term (>18 mo) requirements for primary research [41]
	Engaging policymakers and stakeholders in the production of reviews and review-derived products [42]
	Engaging policymakers and stakeholders in the “merit review” of reviews and review-derived products
Packaging reviews and review-derived products for policymakers and stakeholders	Wording the title in a way that would engage policymakers and stakeholders (e.g., as a question, with a solution-orientation)
	Presenting findings using an “inverted pyramid” (e.g., bulleted key messages, executive summary, full report)
	Highlighting the take-home messages from the review, particularly decision-relevant information (e.g., benefits and harms of policy options)
	Highlighting the contexts in which the included studies were conducted that might influence assessments of local applicability
	Highlighting the characteristics of the participants in the included studies and the contexts in which the studies were conducted that might raise equity considerations
	Using a format that is consistent, visually interesting (e.g., a mix of colours and of bulleted text, figures, and tables), and “skimmable”
	Using language that is appropriate to policymakers and stakeholders, with technical language restricted to an appendix
Disseminating reviews and review-derived products to policymakers and stakeholders	Providing an option to sign up for an e-mail alert when new reviews or review-derived products are posted online
	Sending a “refresher” e-mail alert about a review or review-derived product when a “window of opportunity” opens
	Engaging policymakers and stakeholders in providing online commentaries about specific reviews or review-derived products
	Providing online briefings (e.g., webinars) about specific reviews or review-derived products
	Providing face-to-face briefings about specific reviews or review-derived products
	Giving presentations at meetings about specific reviews or review-derived products coupled with policymaker commentaries
	Executing proactive knowledge-translation strategies (e.g., identifying key messages, determining the principal target audiences for each of these messages, seeking out the most credible messenger for these messages and engaging their interest in becoming involved in the communication of these messages, and supporting their communication efforts) [43]
Providing policymakers and stakeholders with the resources, skills, and opportunities to find and use reviews and review-derived products when they need them	Making reviews and review-derived products available through policymaker-targeted, searchable databases [44] (e.g., PPD/CCNC database for health system-targeted interventions, Rx for Change database for clinician-targeted interventions, and Resource Bank for consumer-targeted interventions)
	Providing policymakers and stakeholders with training so that they can find and use reviews and review-derived products and understand their value relative to other sources of research evidence [45]
	Organizing “deliberative dialogues” at which the knowledge arising from systematic reviews can be combined with the tacit (i.e., how to) knowledge and other types of knowledge brought forward by participating policymakers and stakeholders (e.g., about on-the-ground realities and constraints, citizens' values and beliefs, interest group power dynamics, and institutional constraints) [6]–[8]

PPD, Program in Policy Decision-Making; CCNC, Canadian Cochrane Network and Centre.

The importance of interactions underpins efforts by some organizations to engage both researchers and policymakers in priority-setting and/or production activities and in deliberative dialogues. The importance of timeliness underpins efforts to create and continuously update databases that provide “one stop shopping” for optimally packaged reviews and review-derived products, as well as efforts to develop capacity among policymakers to find and use research evidence efficiently (which includes improving their understanding of how many types of systematic reviews are needed to inform any given policymaking process and raising their awareness about the sources of these reviews and review-derived products). The importance of an accordance between research evidence and policymakers' beliefs, values, interests or political goals, and strategies underpins efforts to identify “windows of opportunity” in policymaking processes and use review-derived products as the basis for engaging policymakers and the stakeholders seeking to influence them, as well as efforts to support the “real time” identification of an accordance (e.g., through well-facilitated deliberative dialogues). However, all of these activities warrant evaluation and, if found to be effective, scaling-up in order to support the use of reviews and review-derived products by all policymakers and stakeholders.

## Conclusion

Supporting the use of systematic reviews in policymaking has received growing attention in recent years. More of the types of reviews needed by policymakers and stakeholders are being produced, which reduces the burden placed on policymakers and stakeholders to find and synthesize the research evidence on their own. Similarly, more review-derived products targeted at policymakers and stakeholders are being produced, and these products increasingly help to address the unique challenges faced by these groups, namely assessing the local applicability of reviews and mobilizing the range of reviews that are needed in different steps in the policymaking process. Finally, many activities to support the use of reviews are being piloted. Future challenges include working through whether and when an apparent duplication of effort in the production of these summaries is problematic and scaling up activities that are found to be effective in supporting the use of reviews and review-derived products.
